# Motion, Relation, and Passion in Brain Physiological and Cognitive Aging

**DOI:** 10.3390/brainsci12091122

**Published:** 2022-08-24

**Authors:** Hermundur Sigmundsson, Benjamin H. Dybendal, Simone Grassini

**Affiliations:** 1 Department of Psychology, Norwegian University of Science and Technology, 7491 Trondheim, Norway; 2 Reasearch Center for Education and Mindset, University of Iceland, 101 Reykjavik, Iceland; 3 Department of Social Studies, University of Stavanger, 4021 Stavanger, Norway

**Keywords:** grey matter, white matter, motion, physical exercises, relation, passion, interest, learning, cognitive ageing, challenges

## Abstract

The aim of the current paper was to present important factors for keeping the basic structures of a person’s brain function, i.e., the grey and white matter, intact. Several lines of evidence have shown that motion, relation, and passion are central factors for preserving the neural system in the grey and white matter during ageing. An active lifestyle has shown to contribute to the development of the central nervous system and to contrast brain ageing. Interpersonal relationships, and interactions, have shown to contribute to complex biological factors that benefit the cognitive resilience to decline. Furthermore, the current scientific literature suggests that passion, strong interest, could be the driving factor motivating individuals to learn new things, thus influencing the development and maintenance of the neural functional network over time. The present theoretical perspective paper aims to convey several key messages: (1) brain development is critically affected by lifestyle; (2) physical training allows one to develop and maintain brain structures during ageing, and may be one of the keys for good quality of life as an older person; (3) diverse stimuli are a key factor in maintaining brain structures; (4) motion, relation, and passion are key elements for contrasting the loss of the grey and white matter of the brain.

## 1. Introduction

The basic elements of a person’s brain function that changes over the brain development are related to the white and grey matter of the brain [1,2]. The grey matter of the brain is composed by several biological structures, such as neuronal cell bodies, dendrites, unmyelinated cell axons, glial cells (astrocytes and oligodendrocytes), synapses, and capillaries. Brain white matter, instead, consists principally of long-reaching myelinated axons [3]. The color difference between these two brain structures is mainly due to the whiteness of the myelin coating the neuron axons.

Several authors have compared the brain to a computer using terminologies borrowed from information technology [4]. From such point of view, it has been sometimes simplified that the grey matter may be considered the processing unit of the brain and the white matter the wires connecting the various parts of the system. The capacity of the nervous system to adjust the organization of the brain structure and function in response to stimuli and experience is called human brain plasticity or neuroplasticity [5]. It may be argued that the neuroplasticity is the mechanism for both development and learning of new skills and knowledge. At the same time, it is the cause of pathology such as, for example, Alzheimer´s disease. This intrinsic property of the nervous system is generally retained throughout a lifespan [6] (pp. 377–378), even though a growing body of literature suggests that factors such as lifestyle and behaviors might significantly affect it [7,8,9].

The brain white matter consists of bundles of axons that are isolated with a layer of lipides (myelin) that carry electric signals between the grey areas of the brain. The myelin layer allows for a fast transmission of the electrical signals through the neuron axons. Fast and effective transmission of the electric signals from one part to another part of the brain is essential to all human cognitive functions and has been shown to be crucial for learning and memory skills [10,11]. Bartzokis [11] argues that both the production and maintenance of myelin is a key factor for typical brain functions. Myelin breakdown may, at the same time, be a causing factor to the pathology of Alzheimer´s disease and ageing. The development of the white and grey matter was studied thoroughly in an important work by Sowell and colleagues [12], examining the lifelong development of the brain structure in individuals aged 7–87 using magnetic resonance imaging (MRI). It has been shown that white matter is such that it grows to approx. 40 years of age and then it decreases [1,12,13]. It has been proposed that the depletion of brain white matter is possibly connected with the tendency of older adults to become cognitively and physically impaired and slower compared to younger people (see e.g., [14]).

It has been empirically shown that the number of neurons composing the grey matter steadily decline starting from the age of ten [12]. Elimination of neurons and synapses in early adulthood via the process of cortical thinning [15] and shrinkage of large neurons [16] or loss of neurons [17] are responsible for the steady decrease in grey matter while aging.

It has been proposed that the reduction in brain grey matter during the lifespan is related to the efficiency of biological systems. One of the most prominent theories attempting to explain the phenomenon is the Neural Darwinism Theory [18,19,20], according to which only those neurons that are used (activated) survive, while the others atrophy and eventually die. During the early phases of the development of the neural system—the childhood—many neurons are available and the plasticity of these neurons to create networks (and therefore to learn) is high. In this phase, the child is generally exposed to a variety of environmental stimuli and learns many basic skills, and such development is accompanied by biological and structural changes in the developing brain. However, with the formation of neural groups, the less efficient neurons and neural groups die. Only the neural groups that are linked in networks with other neural groups survive this selection, and, therefore, the brain neurons tend over time to be selected for their ability to be inter-connected despite reducing the total number of neural cells. This phenomenon could explain why grey matter is reduced over time, and why empirical functional MRI (fMRI) studies have repeatedly shown that older adults activate, to perform the same task, a larger number of neurons compared to younger people [21,22,23]. The potential principle of neurons elimination based on use is especially important in older people [24]. Older individual exhibit less behavioral and neural plasticity compared to younger individuals [25,26]. Even though the brain of most older adults shows some degree of grey matter loss over time, the size of such change greatly varies between individuals, and medically and cognitively healthier individuals have been found to display a lower degree to brain atrophy compared to less healthy individuals [27]. Such evidence has been used to argue that age alone is not the sole factor defining the development of the brain when aging. It has been experimentally observed that fine motor task and gross motor task performances correlate more in older age groups compared to young [12]. It has been proposed in the scientific literature that this could be one of the reasons why there is a higher relationship between two similar tasks when you are older than when you are younger [14]. As a smaller number of neural networks become available to perform a cognitive or motor task, the human brain uses the same networks when similar tasks are performed. Following the comparison between human brain and computers introduced before, it can be said that with increasing age, the computational power is reduced, with the result of a decline in cognitive and motor skills.

Lifestyle is one of the risk factors for cognitive impairment in normal ageing and neurodegenerative diseases [28,29]. Decreased plasticity and resilience such as in increased neurodegeneration and decreased neuroprotection is another risk factor for the development of cognitive dysfunction [29,30,31]. Research also indicates the important role of diet, endogenous metabolic factors, and emotional stimuli in aging of cognitive and motor functions [32,33,34].

Recent research shows that the most important factor in maintaining the grey and white matter is to make use of brain diverse abilities, and, therefore, to frequently electrically engage brain networks related to this activity. Among the human faculties that have been mostly studied for maintaining a healthy brain during aging are physical exercise regularly, i.e., motion [35,36,37,38], maintain strong relationships with friends or family, i.e., relation [39,40], and learn new things or acquire intellectual challenges, i.e., passion [18,19,24,41,42,43]. In the present article, we will review the evidence available in the scientific literature about the relationship between brain structural physiology development in relation to motion, relation, and passion.

## 2. Motion

Brain ability to adapt to internal or external conditions is often referred to as brain plasticity. This brain capability to adapt depends on the capacity of the neurons to modify connections and strength relationships between them on both a local and global level within the brain. Long-term potentiation of the efficacy of communication at the synaptic level is one of the physiological bases of training and learning. This phenomenon can be determined by both presynaptic and postsynaptic mechanisms, and several of these mechanisms have been identified as related to physical activity (see [44]), some examples are: calcium waves due to calcium-induced calcium release (CIRC), from the endoplasmic reticulum [45,46], retrograde transport of proteins [47], mRNA-protein complexes formation and their microtubule-mediated trafficking [48]. These proteins regulate the signaling of synapses to nervous cell nuclei and may be crucial for mediating the activity of the synapses and resulting in the gene expressions that are associated with learning and memory [44]. In line with this idea, a reduced function of the signaling proteins may be associated with brain impairment associated with cognitive problems, psychiatric conditions, and degeneration of the brain tissues [47,49]. In contrast, it has been proposed that an increase in the functions of these proteins could enhance brain function and plasticity, and physical activity may help this process [44].

Other mechanisms that have been suggested to link brain physiological development and physical activity, are brain tissue genesis in reaction to motion, such as gliogenesis, angiogenesis, and synaptogenesis [50].

Furthermore, it has been indicated that physical exercise may increase vascularization of grey matter, as well as the myelination and axonal development in white matter [51].

Several lines of evidence argue in favor of the idea that a positive effect of motion on brain physiology exists [38,52]. Published research has shown that very diverse types of physical training, such as, for example, juggling training [53], mindfulness body–mind training [54], ballet dance training [55], and gymnastic training [56] can all affect brain structure in both grey and white matter. Observational studies have shown that an active lifestyle is helpful for maintaining cognitive and neurological health for all the age groups [57,58]. Greatest effects have been found for higher order processes, such as switching between tasks, working memory and cognitive inhibition [59].

It has been shown that brain white matter in children is more developed for those that exercise more [60], and elderly that exercise more maintain a higher brain functionality compared to those that are less physically active [59]. Cabeza et al. [22] found that active seniors use a larger portion of their brain to solve different tasks than seniors who are less active.

Several intervention studies have confirmed these findings. Aerobic training for six months in a sample of elderly participants (consisting of 1 h of exercise to be performed three times each week) was shown to be effective in increasing grey and white matter [59]. Another study in elderly participants found increased functionality in brain areas related to attention, and regions of the brain related to attention control [61]. The results of these studies provide strong evidence of the role of motion, that is, cardiovascular fitness training, in maintaining and enhancing the structural physiology and functionality of the central nervous system (see [52]).

Recent lines of evidence have suggested that physical exercises influence cognitive performance of elderly individuals on multiple levels, which effectively and sustainably enhance the individual cognitive reserve and show transfer to daily life activities [38]. Enhanced release of brain-derived neurotropic factor with physical activity may be of key importance in this respect [38,62,63].

## 3. Relation

Studies have indicated that good social relations may inhibit cognitive decline and build cognitive reverse directly and indirectly through various mechanisms [64]. A large number of social ties such as friends, family, and neighbors and their engagements, increase complexity and mental stimulation. Maintaining these relations and creating new ones requires effort and skills. Social relations may therefore enhance cognitive reverse through cognitive strategies, greater neural growth, and synaptic density, which protects against pathological processes [65,66]. Studies suggest that people with poor relationships have poor cognitive functions later in life [67,68,69]. However, there are some inconsistencies in findings as other studies have indicated no relationship between social relations and cognitive function later in life [70,71,72]. Supporting the importance of social relations to cognitive reverse, empirical studies that use magnetic resonance imaging, voxel-based morphometry, and gross stereological analysis have demonstrated that more complex and larger social networks are related to a larger volume of the orbitofrontal cortex and the amygdala. Furthermore, a greater amount of white matter lesions has been observed among people who are more socially inactive [73,74]. Furthermore, more grey matter density in brain regions, such as the subgenual anterior cingulate cortex and ventromedial prefrontal cortex, has been related to larger social networks [73].

Randomized control trials (RCT) demonstrate that social relations may enhance cognitive reverse [52,75]. Interventions that aim to increase social relations in community dwelling people have shown benefits to cognitive functions and brain volume compared to control groups [76,77,78]. Furthermore, it has been argued that interventions that aim to enhance cognitive and physical functions and have shown benefits to cognitive function, also involves social relations [77,78]. Thus, the social elements of the interventions could have contributed to the improvements in cognitive functions [70,79].

Personality traits have been shown to be associated with brain grey matter volume. Personality traits supporting human interactions as extraversion have been positively associated with the development of brain regions involved in social cognition and affective process to be associated with personality traits in young adults [80], while personality traits related to antisocial behavior have been shown to be negatively related to grey matter volume in the prefrontal cortex [81].

Based on the accumulated evidence, it is argued that good social relations are important for cognitive reverse and prevent cognitive decline [40]. However, due to methodological issues such as distinct definitions, measurements, and ambiguity in specific contributors to cognitive functions, more robust evidence (e.g., RCT studies) is needed to demonstrate causality.

## 4. Passion

Exercising your mind is important to maintain and establish new neural networks or stronger connections between existing neural connections [19,24]. However, keeping your mind active is not done automatically. Based on the principles of neural plasticity, the extension and formations of new synapses is a result of an active mind [24,82]. Research indicates that both younger and older adults show improvements with memory training. However, the effects seem to be quite specific [83]. Recent research demonstrates that greater involvement in serious leisure in older adults turns into greater levels of subjective well-being and harmonious passion [84].

Furthermore, passion is defined as “a strong feeling toward a personally important value/preference that motivates intentions and behaviors to express that value/preference” [85] (p. 9981). An individual’s passion towards a certain theme, topic, ability, or activity is important in maintaining an active mind. Working with and practicing in the activity through deliberate practice is important, but also relatable to principles of neural plasticity (i.e., use it or lose it, repetition; [24,86]). Former studies have shown that passion is related to more deliberate practice among football players and related to better well-being and better performance among workers [85,87,88]. Based on these findings, it can be argued that passion is important in maintaining principles of neural plasticity, i.e., the passion circle (see for overview [43]). Hence repetition, use it or lose it, use it and improve it and intensity [24]. An example could be learning a second language, which is important for grey matter [37,89]. Being passionate about language and acquiring new languages could motivate the individual to spend more time practicing a second language and thus strengthen the grey matter, the neural cells, and their connections [19]. Furthermore, studies have shown that harmonious passionate individuals have more positive experiences learning a second language [90]. As a result, individuals with passion for language could engage in more deliberate practice, which is closely related to the principles of neural plasticity [24,91]. Strong interest “passion” may be considered the key factor for taking new challenges, learning new things, and the necessary amount of training and repetition needed for developing the skill or knowledge [42,43,86,92,93,94,95].

Furthermore, psychological traits associated with passion—such as grit and growth mindset—have shown to be related to the development of the grey matter in various parts of the brain [96].

**Figure 1:**
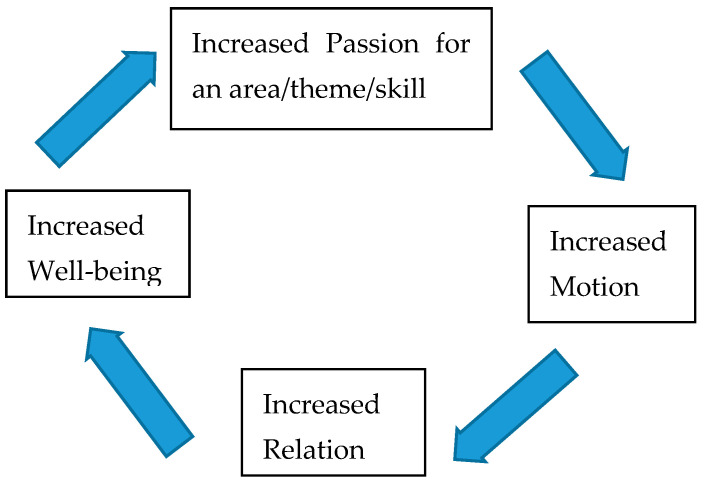
Passion, motion, relation, well-being circle.

It is worth noting that recent research indicates that impaired motor function, antisocial behavior, depression, and anhedonia are common prodromal presentations of neurodegenerative and psychiatric disorders as well as normal ageing [97,98,99,100]. It could be looked upon as a vicious circle. Less passion less motion may be promoting less relation and less well-being. Sigmundsson et al., [92] argue that passion for area, theme, and skill is a basic motivational factor together with grit and mindset. Passion gives direction to the area of interest, which could be related to the dopamine system [101], which is central in attention, learning, goal-directed behaviors, and rewards [94]. Passion may be providing the focus essential for long-term goal achievement [92] (see Figure 1).

## 5. Limitations and Future Directions

We believe the present article summarizes quite an extensive body of literature and proposes an interesting overarching perspective on how different lifestyles and individual differences may be responsible for optimally developing and maintaining key brain structures. However, our study—due to its general and inclusive focus—does not systematically scrutinize the vast body of literature on different lifestyles and brain structure development and ageing. Future theoretical studies should attempt to summarize the existing literature in a further comprehensive and systematic manner.

Future studies should empirically test how motion, relation, and passion may be causally linked to brain development and a favorable ageing process of brain structures. These studies should attempt to obtain high-quality scientific evidence, e.g., employing RCTs.

## 6. Conclusions

The key message in this theoretical perspective article is that we can have an effect on our brain development and ageing. In this context, it is important that our brain functions are continuously trained. Motion (physical exercise), relation (social interactions), and passion (learning new things) are key elements for contrasting the loss of the grey and white matter of the brain.
